# Headache Specialists’ Perceptions of the Role of Health Psychologists in Headache Management: A Qualitative Study

**DOI:** 10.7759/cureus.56175

**Published:** 2024-03-14

**Authors:** Stanley Curtis Takagishi, Amy S Grinberg, Hayley Lindsey, Roberta E Goldman, Sean A Baird, Laura Burrone, Jason J Sico, Teresa M Damush

**Affiliations:** 1 Headache Centers of Excellence (HCoE) Research & Evaluation Center, Veterans Health Administration, Department of Veterans Affairs, Orange, USA; 2 Psychology, James A. Haley Veterans' Hospital, Tampa, USA; 3 Neurology, VA (Veterans Affairs) Connecticut Healthcare System, West Haven, USA; 4 Pain Research, Informatics, Multi-morbidities, and Education (PRIME) Center, VA (Veterans Affairs) Connecticut Healthcare System, West Haven, USA; 5 Anthropology & Family Medicine, Warren Alpert Medical School of Brown University, Providence, USA; 6 Social & Behavioral Sciences, Harvard T.H. Chan School of Public Health, Boston, USA; 7 Health Research & Development Service, Richard L. Roudebush VA (Veterans Affairs) Medical Center, Indianapolis, USA; 8 Neurology, Yale School of Medicine, New Haven, USA; 9 Center for Health Services and Outcomes Research, Indiana University School of Medicine, Indianapolis, USA; 10 Center for Health Services and Outcomes Research, Regenstrief Institute, Inc., Indianapolis, USA

**Keywords:** qualitative methods, behavioral interventions, headache psychologist, health psychology, psychology, headache

## Abstract

Background

Since headache specialists cannot treat all the patients with headache disorders, multidisciplinary teams that include health psychologists are becoming more prevalent. Health psychologists mainly use a form of cognitive-behavioral therapy (CBT), along with biofeedback on occasion, to effectively address patients’ pain and headache disorders. The Veterans Health Administration (VHA) is one setting that routinely includes a health psychologist with advanced training in pain disorders in their pain care to its veterans. The VHA has established Headache Centers of Excellence (HCoE) around the country to provide multidisciplinary treatment for patients with headache disorders, which enables headache specialists to regularly interact with health psychologists.

Objective

The study’s objective is to evaluate headache specialists’ views of health psychologists in the treatment of patients with headache disorders.

Method

Semi-structured interviews were conducted with headache specialists in academic-based healthcare settings, the community, and VHA HCoE sites. The interviews were audio-recorded and de-identified so they could be transcribed and analyzed using content matrix analysis.

Results

Four themes emerged: headache specialists desired to work with health psychologists and included them as members of multidisciplinary teams; valued health psychologists because they provided non-pharmacological treatments, such as CBT and biofeedback; preferred in-person communication with health psychologists; and used multiple titles when referring to health psychologists.

Conclusion

Headache specialists valued health psychologists as providers of behavioral and non-pharmacological treatments and considered them essential members of multidisciplinary teams. Headache specialists should strive to work with a headache psychologist, not just a general health psychologist. By committing to this, headache specialists can foster changes in the quality of care, resource allocation, and training experiences related to health psychologists.

## Introduction

In the United States, severe headaches or migraines are self-reported by 21% of females and 10% of males, while tension-type headaches are experienced by 42% of females and 36% of males [1-3]. The disability from these headache disorders affects not only the individuals [4,5] but also their employers and the healthcare system [6,7]. Since headache specialists have been unable to adequately meet the national need to treat all the patients with headache disorders [8,9], headache specialists have increasingly supported a multidisciplinary approach in the treatment of patients with headache disorders [10].

Multidisciplinary headache care is often proposed to involve coordination between a headache specialist and many other providers [11] but is most effective when the care amongst the disciplines is considered comprehensive by the multidisciplinary team [12]. One provider that is routinely mentioned as part of these multidisciplinary teams is a health psychologist [12]. However, within the field of health psychology, headache care is not yet an established specialty, even though there has been a growing emphasis in the treatment of patients with pain disorders to utilize health psychologists to decrease the impact that pain has on patients’ functioning and psychological well-being [13]. Health psychologists use their skills to promote the health of the individual, prevent illness, and improve the effectiveness of healthcare systems [14]. Healthcare organizations employ health psychologists, when they are available, to specifically treat chronic pain conditions and associated comorbidities, to instruct patients on active self-management strategies, and to act as important members of multidisciplinary care teams by facilitating the development of fully effective treatment plans with other clinical providers in primary and specialty care settings [15].

Health psychologists who work primarily with patients with pain disorders are more prevalent within the Veterans Health Administration (VHA) than in other healthcare organizations [16]. The VHA has made an effort to expand pain management treatment to its veterans after VHA Directive 2009-053, which formally established a Stepped Care Model for Pain Management [17]. This pain care framework was founded on a biopsychosocial, patient-centered philosophy offered by a team of professionals, which routinely included a health psychologist with an advanced understanding of pain disorders.

In addition to offering more general pain care to its veterans, since 2018, at the behest of Congress, the VHA has established 19 Headache Centers of Excellence (HCoE) sites around the United States to provide comprehensive, multidisciplinary treatment for veterans with specific pain in the head region or with headache disorders. This was driven by the need of the large numbers of veterans who presented to VHA with headache disorders. Of the nine million veterans who received care in the VHA each year, approximately 14% had a diagnosis of headache, amounting to 1.44 million veterans in the 2019 fiscal year [18]. Similar to the general population, an overwhelming majority of veterans with headache diagnoses had been treated only within a primary care setting, although many would benefit from care provided by headache specialists and a multidisciplinary team [18].

Due to the large number of patients with headaches across the United States both within the general population and the VHA, headache specialists commonly refer patients with headache disorders to health psychologists for assistance in the treatment of primary (e.g., migraine and tension-type) and secondary (e.g., post-traumatic and cervical) headache disorders. However, the reasons why headache specialists regard health psychologists as regular referral recipients in the provision of headache treatment have never been explored in detail through direct individual interviews. The specific aim of this paper is to elucidate headache specialists’ views on the desired role of health psychologists in the treatment of headache disorders in collaboration across care settings.

## Materials and methods

Recruitment of subjects

We contacted headache specialists in academic-based healthcare settings, non-university healthcare organizations, as well as VHA specialists providing headache care at the existing VHA HCoE sites. For VHA HCoE provider recruitment, the clinical directors at each of the HCoE sites were contacted by email to request interviews with their headache specialists. The National Veterans Affairs (VA) HCoE Director sent invitations to the non-VA providers asking for their participation.

We sought only the opinions of headache specialists. We interviewed a provider as a headache specialist if they met at least two of the following criteria: actively treating patients within a VHA HCoE; practicing in a US-based, non-VHA headache center; experienced in establishing, developing, and maintaining a headache center; involved in national headache professional associations; and having published academic papers on headache neurology topics during their career.

Standard protocol approvals, registrations, and provider consent

The study was approved by the VHA Connecticut Healthcare System Institutional Review Board, and this project followed the Consolidated Criteria for Reporting Qualitative Studies qualitative research guidelines [19].

Participants consented verbally to participate and allowed audio recordings of their interviews. Participation was voluntary and the data were de-identified.

Interview

The interview guide was developed specifically for this study by members of the study team (JJS, TMD, HL, REG) based on a review of the literature and the team’s experience within this area of study. The interview guide used predominantly open-ended questions to explore topics relevant to the establishment, need, and implementation of headache services. Study team personnel (HL, JJS, TMD, REG) with either a BA, MD, or PhD had prior experience conducting semi-structured interviews and were trained to use the developed interview guide. Audio-recorded interviews lasting 30 to 60 minutes were conducted on-site at the participant’s facility and telephone interviews occurred for all other VHA clinicians and non-VHA clinicians.

Data collection

We conducted qualitative, semi-structured interviews over 15 months from January 2019 through March 2020. The goal was to inquire about a wide variety of topics during each interview and to gather additional information if specific areas were mentioned. Hence, the semi-structured interview included the standard prompt: “What types of treatments are offered within your center?” Whenever the terms psychology or health psychology were mentioned in this answer, additional inquiries specifically crafted to understand perceptions about the role of psychology and health psychology were made, such as: “How is psychology or health psychology currently used?”; “How will their use be expanded?” In addition, all the interview data were examined to determine if these providers or any other providers mentioned psychology or health psychology when answering any question that was asked, and these data were also included for analysis.

Data analysis

Interview recordings were transcribed, de-identified, and imported into NVivo 12 software (QSR International, Melbourne, Australia) [20]. Team members (TMD, HL, REG) created a codebook with code definitions after analyzing the interview guide and a selection of transcripts. They tested, revised, and clarified the codebook as necessary. Four team members (SB, SR, HL, LB) coded the transcripts, with two coders assigned to each transcript. The two coders independently read each transcript, assigned codes within NVivo to data segments, then discussed any discrepancies to come to a consensus and merged the files for further data analysis. The specific identifiers given to each headache specialist that is quoted are listed in Appendix A.

Next, the analysis team conducted a content matrix analysis using the codes “Psychology” and “Health Psychology” to determine the data used in this study. Through evaluation of the data, themes regarding the subjects’ perceptions of the role of a health psychologist in the delivery of headache care were identified. Finally, the titles that headache specialists used when referring to a health psychologist were examined.

## Results

Participants

The responses of 25 headache specialists were examined after the mentioning of psychology or health psychology to the standard prompt, and if psychology or health psychology was cited in any answer during the full interview. A more in-depth discussion of the role of health psychologists in the management and treatment of patients with headache disorders occurred when the follow-up prompts were voiced.

Thus, an analysis was conducted on the data from the following headache specialists: 18 neurologists, two physiatrists, three nurse practitioners, one registered nurse, and one occupational therapist. Regarding the headache specialists, 17 were females, eight were males, 16 were VHA, nine were non-VHA, and the average years of experience treating headaches was 13.7 years for VHA, 20.8 years for non-VHA, and 16.6 years of experience for all of the interviewed headache specialists (years of experience information was missing for three VHA specialists).

Headache specialists’ views of health psychologists

Four themes arose regarding headache specialists’ views on the role of health psychologists: (1) headache specialists desired to work with health psychologists and regularly included them as members of multidisciplinary headache treatment teams; (2) headache specialists valued health psychologists as providers of non-pharmacological treatments such as cognitive-behavioral therapy (CBT) and biofeedback; (3) headache specialists preferred in-person communication with a health psychologist; and (4) headache specialists used multiple titles when referring to health psychologists.

Headache Specialists Desired to Work With Health Psychologists and Regularly Include Them as Members of Multidisciplinary Headache Treatment Teams

The headache specialists interviewed considered a treatment setting to be better equipped to treat individuals with headache disorders if they had a health psychologist employed as a staff member.

We’ve had some challenges in terms of being able to find an appropriate headache psychologist to be a part of our program. I’m hoping that’s a temporary issue; and certainly that’s a necessary integral part of any headache specialty center. (200 Non-VA Provider 02)

Headache treatment settings with a health psychologist are viewed as “more specialized headache clinics” (102 VA Provider 01).

Headache specialists asserted that health psychologists are extremely important colleagues for providing treatment to their headache patients. According to the headache specialists who were interviewed, health psychologists assisted their patients in multiple ways, such as reinforcing medication recommendations.

Pain psychology. Yes, I think that is a very important part…in terms of a policy of reinforcing minimization of opioid use specifically as one the big challenges in pain medicine, and yes, I do. I think that extending this would be very helpful. (110 VA Provider Focus Group 01)

Hence, whenever a headache specialist described a multidisciplinary team to care for headache patients, a health psychologist was included: “We had a multidisciplinary clinic every other week that would have a psychologist there”; “Embedded within the comprehensive pain center, that’s where I have pain psychology”; and “I think that we have multidisciplinary ideas, but a multidisciplinary clinic is a different thing, and it’s a different beast at the VHA. Pulling in psychology there. Either in the same location or on the same visit” (106 VA Provider 05).

One headache specialist described health psychology as a founding discipline for a multidisciplinary headache program that has been included within a VHA HCoE.

When we started the [Multidisciplinary outpatient headache program], it was me and psychology and OT. (101 VA Provider 01)

The headache specialists who do not have a health psychologist working in their medical center had strong opinions about the need for their patients to have access to health psychology and the difficulty of hiring these specialty psychologists. When asked, “Do you have health psychology services at your center?”, one response was, “Boy oh boy, do I wish that I could say yes to that question” (200 Non-VA Provider 11). Another said, “Pain psychology is, it’s hard to get a psychologist…They’re not growing on trees, and pain, oh, it’s such a hot button issue” (106 VA Provider 02).

Participants explained that at times it was easier to train a psychologist to effectively treat patients with headaches than to search for a health psychologist.

I think we have another resource for psychology. It’s just basically training, having them trained…really focus on headache management and some other things that are new in terms of psychology, dealing with patients with headaches. (112 VA Provider 01)

Headache Specialists Valued Health Psychologists as Providers of Non-pharmacological Treatments Such as CBT and Biofeedback

Headache specialists consider health psychologists to be providers of treatments that do not involve medications. One headache specialist stated, “I mean we’re trying to make more connections with pain psychologists and trying to provide more non-pharmacologic interventions” (109 VA Provider 01). Specifically, headache specialists perceived health psychologists as the best providers to offer CBT. When working with a health psychologist, headache specialists were confident that their patients would have CBT available to them.

If I just can see that somebody’s really gonna need some psychology care, cognitive behavioral therapy, and I refer that right away.” (200 Non-VA Provider 08)

Headache specialists were also aware that health psychologists provided biofeedback. Since biofeedback is viewed as an effective treatment by most headache specialists and patients with headache disorders are familiar with the treatment, using the specific term has helped headache providers get patient buy-in for treatment with a health psychologist.

Just increasing our connections with the pain psychologists. We just made a great connection with her because she started her own headache group for biofeedback…and that label seems to be the key to get people to agree to do it. And you know, kind of an end run around the psychology problem of referral and all the concerns patients have that you’re gonna label them as crazy or whatever.(109 VA Provider 01)

Headache Specialists Preferred In-Person Communication With a Health Psychologist

Headache specialists emphasized their preference for direct and in-person communication.

They’re right down the hall from me, and I can refer to them….We also have the luxury of seeing each other in the hallway and discussing cases and kind of deciding, person-to-person what to do. (200 Non-VA Provider 09).

Another specialist commented:

It is basically an open-door policy. Like if I feel at any point that I need help with any of my patients, especially with psychology in case that I encounter some behavioral problems, he is very available for me. So, it is like I can call anybody, and someone would be there helping us. (101 VA Provider 03)

Headache specialists stated that it is easier for their patients to accept psychological treatment when the health psychologist has a physical presence in the same clinic. Referrals are facilitated by the headache specialist being able to personally introduce the patient to the health psychologist, which is considered “the best level of care” (101 VA Provider 01).

It is a direct communication. We even see the patients together, which is a huge benefit. I do my part, and then I turn to him and say, ‘Now, you help with this.’ So, the same thing would be ideal with headache patients. You’re having stressors or psychosocial issues. I’m only going to get so far with this medication. So, if I could turn to somebody right here, or walk you right next door, and say ‘Help me out here. Here is what we’ve dealt with. You do it. (106 VA Provider 05)

Headache specialists noticed when communication between them and health psychologists does not flow easily due to not being co-located: “I do wish that it was more of a streamlined connection between our two departments” (200 Non-VA Provider 07).

Headache Specialists Used Multiple Titles When Referring to Health Psychologists

In our study, headache specialists referred to health psychologists using various titles. Four of the headache specialists used the title health psychologist during their interviews. Ten of the headache specialists used the title pain psychologist, and one of the headache specialists used the title headache psychologist. The remaining 10 of the headache specialists used the generic title psychologist.

## Discussion

This current study examined how headache specialists viewed the role of health psychologists in the treatment of patients living with headache disorders. Overall, this study found that headache specialists recognized the importance of the field of health psychology and included health psychologists in their multidisciplinary headache teams to provide essential, behavioral headache treatments to their patients. However, the headache specialists seemed to use multiple titles for health psychologists who possess headache-specific training and expertise.

The headache specialists interviewed supported the long-standing belief that psychological interventions can effectively aid patients with headache disorders by expressing their desire to work with health psychologists [21]. Health psychologists researching effective headache management strategies would seem to be ideal colleagues for headache specialists who are attempting to establish a specialized headache clinic or a center of excellence [22-25]. However, the lack of available health psychologists, cited in this study by headache specialists, indicated there is a need for more health psychologists to be trained in this area, and there is an obvious opportunity for interested health psychologists to be valued as experts by the population of patients with headaches, which appears to be large and underserved.

An explanation for the dearth of professionals available is that the field of health psychology has just started to establish formal definitions and training criteria to delineate the area of pain psychology and the required expertise to be an effective pain psychologist [26]. Due to the multidimensional nature of this problem, it is well-known among health psychologists who work with patients with pain disorders that it is necessary to develop competency in a core set of skills (e.g., understanding the biopsychosocial nature of pain, proficiency in implementing evidence-based psychological treatments for pain, and understanding how cultural context affects the experience of pain) to successfully treat patients with pain problems [27]. Further discussion is required to determine the specific skills needed for health psychologists to best help patients with headache disorders. Therefore, a headache specialist who values the skills of a health psychologist should not only promote the specialization of pain psychology within the field of health psychology but also encourage defining the specific qualifications necessary for health psychologists to most effectively treat patients with headache disorders.

Patients with headaches, and especially patients with chronic headaches, present to providers as patients with a refractory pain disorder, with multiple diagnosed co-morbidities, and whose problem may best be managed by a multidisciplinary team [12,28,29]. Multidisciplinary care for patients with headache disorders is increasing in prevalence and could soon be proposed as the standard of care [10,30]. The interview data reported in this current study provide additional detail on the thoughts of headache specialists related to health psychologists regarding the specific training and skills that are most valued during their collaborative treatment, specifically behavioral treatments such as CBT and biofeedback.

Interestingly, addressing psychological comorbidities, such as psychosis, mood deficiencies, or personality disorders, was not cited by the headache specialists in the results of this research. For some headache specialists, psychologists are only consulted to address significant psychological co-morbidities [31]. Headache specialists seem to be realizing that many of their patients benefit from or prefer non-pharmacological interventions [32,33], even though most of the headache specialists interviewed are medication prescribers. Health psychologists’ knowledge of behavioral treatments can complement the expertise and services of other providers creating a healthy mix of skills, which is one of the principles of an effective multidisciplinary team [34].

Headache specialists valuing a health psychologist’s ability to effectively provide CBT for their headache patients provided a further endorsement of CBT being able to produce positive results [35-37]. Hence, a manualized protocol designed to implement CBT for patients with headaches would likely be highly supported by the interviewed headache specialists [38].

Biofeedback treatment for patients with headache disorders also would appear to have the endorsement of headache specialists, even though current published research in this area is lacking [39-42], or applies mainly to children [43,44]. This suggested that present-day research confirming the effectiveness of biofeedback training for headache disorders needs to be conducted.

Furthermore, the headache specialists interviewed seemed to describe how communication that occurs in person is regular and readily available due to proximity fosters an improved relationship between themselves and health psychologists. They believed that treatment is improved through this enhanced relationship when a verbal exchange occurs directly, physically face-to-face, and in a timely manner about the proposed treatment plan, rather than having information communicated through electronic records that may have been crafted several days or weeks in the past. Better communication among team members can produce improved coordination of care, a more appropriate plan of treatment, and better outcomes for the patient [45-47]. Comprehensive care is generally facilitated when a “warm handoff” occurs that allows personal contact between team members [48,49]. Headache specialists appeared to state that a higher and more effective level of care is more likely to be possible when the health psychologist is physically present in the same healthcare setting.

Finally, headache specialists seemed to use multiple titles during their interviews in this study to refer to health psychologists. The field of health psychology provides psychologists an area of focus to seek out additional professional training to help patients with medical problems, such as pain and headaches [31]. The majority of headache specialists, who were mostly neurologists, referred to psychologists with a descriptor such as health, pain, or headache (e.g., health psychologist, pain psychologist, and headache psychologist). By using modifiers, the data could be interpreted that these specific headache specialists indicated that specialization is desired for the psychologists who will be treating their patients.

Nonetheless, the other headache specialists interviewed, i.e., 10 of the 25, used only the general term, psychologist. It is notable that such a large number of headache specialists interviewed did not identify the psychologist working in a multidisciplinary headache care team with a specialized title designating them as an expert with relevant, increased knowledge about pain or headache disorders. This lack of consistency was a surprising observation and indicated that one title should be established so all headache specialists will refer to health psychologists who are experts in treating patients with headaches in a standardized manner.

Headache psychologist was a title used by a headache specialist interviewed in this study. The title of headache psychologist describes an additional level of specialization beyond health psychology and identifies a specific knowledge and skill set within the area of pain psychology. We propose the title of headache psychologist accurately reflects the ideal type of psychologist to provide treatment for patients with headache disorders. The type of training and experience for a psychologist to qualify as a headache psychologist has not been outlined in any official guideline. However, it is submitted here that a psychologist who desires to be a headache psychologist would first focus their general training in the area of health psychology, then obtain the specialized clinical skills necessary to be a pain psychologist, and finalize their experiences by becoming proficient treating patients with the primary pain complaint of headache. A future research project could determine the more specific qualifications required to be designated as a headache psychologist.

The utilization of an accurate title for headache psychologists is important for providing services in a multidisciplinary headache center because using a professional title is shorthand for describing to the patient the expertise and set of responsibilities possessed by a provider. A title communicates the specialized knowledge, competencies, training, and experience that a person who holds the title is likely to possess [50]. The title used sends a clear signal about a healthcare provider’s role and identity to the patient because it conveys a meaningful status signal when employed explicitly and consistently [51]. An accurate title also helps providers channel their attention and energy more effectively in their professional development, have higher-quality interactions with other professionals, and better utilize their capabilities in their work [52].

Once headache specialists employ the title of headache psychologist routinely, they could strive to state directly that a headache psychologist should be involved in all settings that treat patients with headaches. This goal would be especially relevant when a headache specialist is choosing a psychologist to be a member of a multidisciplinary headache treatment team. Also, the headache specialist could use this information as a rationale for additional funding from the medical system that may be needed to employ or recruit a headache psychologist, who by the proposed designation, has more specialized knowledge and experience. Similarly, a headache specialist could substantiate the need for a general health psychologist on a treatment team to receive or be provided more advanced training with patients who have headache disorders to develop the level of expertise to qualify as a headache psychologist.

Limitations

Limitations of this study include that this analysis was part of an extensive interview on a multitude of topics limiting the time spent by respondents on this topic. Nonetheless, we sampled headache specialists across healthcare systems to elucidate their perceptions of the role of the health psychologist in headache care. Future research may evaluate further the precise titles of employed psychologists working in multidisciplinary headache teams across healthcare systems.

Another limitation is the interviews were conducted prior to the COVID-19 pandemic. Headache specialists indicated their preference for in-person communication with health psychologists; however, these responses may be different if discussed today. Also, it is possible that in-person communication is preferred by headache specialists with all providers, especially those who are co-located in close proximity and not just with health psychologists. Determining how headache specialists prefer to communicate with health psychologists and other providers after changes that have occurred in the work environment due to the COVID-19 pandemic should be clarified with future research studies.

## Conclusions

Headache specialists interviewed for this study expressed greatly valuing the contribution of health psychologists in providing headache treatment, especially as providers of non-pharmacological treatments such as CBT and biofeedback. Since headache specialists alone are unable to provide comprehensive care to a large number of patients with headache disorders, headache specialists describing their desire to practice collaboratively with health psychologists is important because it emphasizes that health psychologists should be routinely involved in the care of this population. Headache specialists in this study consider health psychologists as essential professionals within specialized headache centers and integral members of a multidisciplinary treatment team. This supports having a health psychologist employed within every setting where patients with headaches are treated. To ensure quality care, however, it is asserted that headache specialists should strive to work with health psychologists who can be identified with the specialized title of headache psychologist. By committing to this goal, headache specialists can foster these significant changes: (1) patients with headache disorders will be assured treatment from a headache psychologist, who has the highest level of skill, expertise, and experience in this area; (2) resources needed to recruit or train headache psychologists can be justified; and (3) health psychologists will be motivated to pursue additional experiences to qualify as a headache psychologist.

## Appendices

Appendix A

**Table 1:** Headache specialists quoted

Code	Specialist(s) – respondent	Setting – VAMC or University Healthcare System
101 VA Provider 01	Neurologist specializing in headache care 01	Veteran Affairs (VA) Medical Center (VAMC) 101
101 VA Provider 03	Occupational therapist specializing in headache care 03	Veteran Affairs (VA) Medical Center (VAMC) 101
102 VA Provider 01	Neurologist specializing in headache care 01	Veteran Affairs (VA) Medical Center (VAMC) 102
106 VA Provider 02	Neurologist specializing in headache care 02	Veteran Affairs (VA) Medical Center (VAMC) 106
106 VA Provider 05	Neurologist specializing in headache care 05	Veteran Affairs (VA) Medical Center (VAMC) 106
109 VA Provider 01	Neurologist specializing in headache care 01	Veteran Affairs (VA) Medical Center (VAMC) 109
112 VA Provider 01	Neurologist specializing in headache care 01	Veteran Affairs (VA) Medical Center (VAMC) 112
110 VA Provider Focus Group 01	Neurologist specializing in headache care 01	Veteran Affairs (VA) Medical Center (VAMC) 110
200 Non-VA Provider 02	Neurologist specializing in headache care 02	University Healthcare System
200 Non-VA Provider 07	Neurologist specializing in headache care 07	University Healthcare System
200 Non-VA Provider 08	Neurologist specializing in headache care 08	University Healthcare System
200 Non-VA Provider 09	Neurologist specializing in headache care 09	University Healthcare System
200 Non-VA Provider 11	Neurologist specializing in headache care 11	University Healthcare System
